# Seasonal Distribution of Psychiatric Births in England

**DOI:** 10.1371/journal.pone.0034866

**Published:** 2012-04-04

**Authors:** Giulio Disanto, Julia M. Morahan, Melanie V. Lacey, Gabriele C. DeLuca, Gavin Giovannoni, George C. Ebers, Sreeram V. Ramagopalan

**Affiliations:** 1 Wellcome Trust Centre for Human Genetics, University of Oxford, Oxford, United Kingdom; 2 Department of Clinical Neurology, University of Oxford, Oxford, United Kingdom; 3 London School of Hygiene and Tropical Medicine, London, United Kingdom; 4 Blizard Institute of Cell and Molecular Science, Queen Mary University of London, Barts and The London School of Medicine and Dentistry, London, United Kingdom; University of Swansea, United Kingdom

## Abstract

There is general consensus that season of birth influences the risk of developing psychiatric conditions later in life. We aimed to investigate whether the risk of schizophrenia (SC), bipolar affective disorder (BAD) and recurrent depressive disorder (RDD) is influenced by month of birth in England to a similar extent as other countries using the largest cohort of English patients collected to date (n=57,971). When cases were compared to the general English population (n=29,183,034) all diseases showed a seasonal distribution of births (SC *p*=2.48E-05; BAD *p*=0.019; RDD *p*=0.015). This data has implications for future strategies of disease prevention.

## Introduction

Seasonality dominates many features of the global environment and that this will impact human health appears inevitable. Seasonal factors can potentially act even before birth, when according to the “fetal origin of adult disease hypothesis“ environmental influences leading to changes in embryonic/fetal tissue structure and function can influence the risk of adult physiological and pathological conditions [1]–[2]. As a consequence, being born in a certain time of the year may influence susceptibility to disease later in life. This is the case for several psychiatric diseases. Most studies performed to date have focused on schizophrenia and data collected on several thousands of patients across different countries suggest an excess of winter and early spring births [3]–[7]. Other psychiatric and psychological traits which have been associated with season of birth include bipolar disorder, major depression and suicidal behavior [4], [8]–[12].

The concept of a season of birth study is simple and involves comparing the season or month of birth of individuals with a condition and comparing them to controls. However, performing the study with scientific rigour is more complicated. For example, since the size effect reported by such studies is often very small a large number of patients is required for statistical power. Furthermore, pooling data from different countries represents another potential source of bias since general population birth trends can substantially differ between countries.

We aimed to further confirm the association of psychiatric disease with season of birth using three new large and recently collected cohorts of schizophrenia (SC), bipolar affective disorder (BAD) and recurrent depression disorder (RDD) patients in a single country (England). To our knowledge this is the largest study performed to investigate this hypothesis within England.

## Methods

Data on month of birth was collected for 57,971 patients from the English Hospital Episode Statistics (http://www.hesonline.nhs.uk/). In particular we gathered information on English resident outpatients seen by a doctor between 2003 and 2011 across England and suffering from SC (ICD-10=F20; n=26,676), BAD (ICD-10=F31; n= 14,569) and RDD (ICD-10=F33; n=16,726). As an additional non psychiatric control we also collected month of birth data from 3,545 Parkinson's disease (ICD-10=G20) patients. To avoid counting the same patient more than once with the same condition, counts were based on the unique patient identifier (HESID) which is based on date of birth, postcode, sex, local patient identifier and NHS number. Patients were compared to 29,183,034 English general population births between 1950 and 1990 from the Office for National Statistics (http://www.ons.gov.uk/). We used the Walter and Elwood seasonality test to estimate within year fluctuations with a 12 month periodicity (simple harmonic seasonal variation) [13]. This test represents a standard technique in the analysis of seasonal trends and has been previously utilized in several month of birth studies [14]–[16]. Monthly odds ratios (OR) were calculated by comparing frequencies of patients and controls born in a certain month vs the rest of the year.

## Results

The birth distribution of controls, SC, BAD and RDD patients is presented in Table 1. In order to assess whether season of birth influences susceptibility to psychiatric conditions we initially compared the distribution of SC, BAD and RDD patients with that of the general population using the Walter and Elwood test. Strikingly the birth distribution of all these conditions was found to be significantly different from that of the general population (SC *p*=2.48E-05; BAD *p*=0.019; RDD *p*=0.015). No difference was observed between patients suffering from Parkinson's disease and the general population (*p*=0.48).

**Table 1:** Number and percentages of psychiatric and general population births.

	SC		BAD		RDD		Controls	
Month	n	%	n	%	n	%	n	%
**J**	2,502	9.38	1,318	9.05	1,455	8.70	2,437,453	8.35
**F**	2,117	7.94	1,154	7.92	1,299	7.77	2,299,108	7.88
**M**	2,299	8.62	1,305	8.96	1,516	9.06	2,615,885	8.96
**A**	2,283	8.56	1,235	8.48	1,462	8.74	2,470,002	8.46
**M**	2,333	8.75	1,351	9.27	1,539	9.20	2,575,367	8.82
**J**	2,184	8.19	1,170	8.03	1,449	8.66	2,452,463	8.40
**J**	2,186	8.19	1,283	8.81	1,388	8.30	2,502,303	8.57
**A**	2,145	8.04	1,141	7.83	1,359	8.13	2,436,238	8.35
**S**	2,235	8.38	1,118	7.67	1,355	8.10	2,424,042	8.31
**O**	2,103	7.88	1,204	8.26	1,350	8.07	2,389,572	8.19
**N**	2,105	7.89	1,134	7.78	1,211	7.24	2,248,217	7.70
**D**	2,184	8.19	1,156	7.93	1,343	8.03	2,332,384	7.99
**TOT**	26,676	100	14,569	100	16,726	100	29,183,034	100

**Figure 1:**
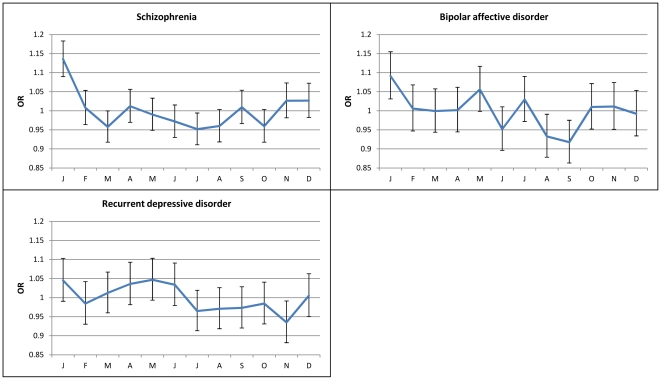
Monthly OR based on month of birth in SC, BAD and RDD.

When monthly ORs were calculated, SC had a statistically significant peak in January (OR=1.13, 95%CI=1.09–1.18, *p*<0.0001) and a significant trough exactly six months later in July (OR=0.95, 95%CI=0.91–0.99, p=0.02). Peak to trough ratio showed a 17% increased risk for January vs July born individuals (OR=1.17, 95%CI=1.095–1.24, *p*<0.0001). Similarly to SC, BAD patients were more likely to be born than expected in January (OR=1.09, 95%CI=1.03–1.15, p=0.002) and less in August and September (OR=0.93, 95%CI=0.87–0.99, p=0.02; OR=0.91, 95%CI=0.86–0.93, p=0.005). The January vs September ratio also indicated a 17% increased risk (OR=1.17, 95%CI=1.08–1.26, p<0.0001). In RDD an almost significant May peak (OR=1.04, 95%CI=0.99–1.1, p=0.08) and a significant November deficit (OR=0.93, 95%CI=0.88–0.99, p=0.02) were observed. Odds ratios with 95% CI are presented in Figure 1.

## Discussion

We report here the largest study performed on month of birth and psychiatric conditions in England. In agreement with previous reports, seasonality of births was detected in SC, BAD and RDD patients. No significant difference was found between Parkinson's disease patients and the general population.

SC births showed the most striking seasonality. These results are also consistent with those of a previous study of English SC patients born between 1921 and 1960 indicating that the season of birth effect is a stable feature of SC [5]. Furthermore, the OR values are surprisingly large and higher than those of a meta-analysis collecting data from more than 126,000 SC patients [3].

Similarly to SC, BAD also appeared to occur more frequently in January born individuals and to follow a seasonal distribution. This is in agreement with a large study of psychiatric patients from the United States in which the risk of both SC and BAD was significantly higher among winter born individuals [4]. This is interesting given the well established overlap between these two conditions. Psychotic symptoms such as hallucinations and delusions are key manifestations of SC but also present in BAD. Furthermore, studies have confidently shown an increased risk of SC in relatives of patients suffering from bipolar disorder and vice versa [17]–[18]. Finally, genome wide association studies have shown the presence of overlap between genetic determinants of SC and bipolar disorder [19].

The risk of RDD was also seasonal but as compared to SC and BAD the effect appeared to be smaller and shifted to later in spring (peak) and autumn (trough). Not many studies have assessed the seasonality of births in patients with depression. However, our results are consistent with those of Torrey et al who reported a spring excess of births in a large cohort of American patients [4]. Furthermore, Salib et al investigated the seasonality of birth of 26,915 British suicide cases and found excess and deficit of births in April–May and October–November respectively suggesting the presence of shared early life factors predisposing to both depression and suicidal behavior [9].

This study has limitations. Information on sex and ethnicity was not available and this represents a potential source of bias. However the large sample size (57,971 cases and 29,183,034 controls), and the strong *a priori* evidence for a season of birth effect in psychiatric conditions makes the data unlikely to be a chance finding. It could be that parents of psychiatric patients have an unusual pattern of conception and it would have been interesting to investigate whether the birth distribution of unaffected siblings of these psychiatric patients also follows a seasonal trend. Unfortunately data on siblings were not available to us, but other studies have tried to answer this question (in particular in SC) and overall the season of birth bias appears present in SC patients but not in their unaffected siblings [20]–[24]. This suggests that the role played by the season of birth in psychiatric conditions is truly causative.

Despite researchers' efforts, the causative factor/s behind this association remains unknown. A key factor which needs to be considered is that the risk is not only higher in certain months of the year vs others but also follows a seasonal distribution. This suggests a seasonal nature of the environmental causative factor. Seasonality dominates global environment, influencing effects of changing climate, sunlight, diet and infection. Interestingly, several lines of evidence now support a role for vitamin D deficiency in the pathogenesis of psychiatric conditions, in particular schizophrenia [25]. Animal models of gestational vitamin D deficiency highlight the important role of vitamin D for correct neurodevelopment [25]. Furthermore, neonatal vitamin D levels are significantly associated with risk of schizophrenia later in life [26]. Finally, the size of the month of birth effect in SC increases with increasing latitude [3]. Similar mechanisms may apply to BAD and RDD, but the evidence for vitamin D in these disorders is much less strong and other factors such as maternal infections and fluctuations in diet may also play an important role.

To conclude, season of birth influences the risk of SC, BAD and RDD in England to a similar extent as other countries and suggests that at least a proportion of psychiatric disease could be prevented by ameliorating the risk factor/s responsible for these intriguing epidemiological observations. It is now paramount to identify the seasonal factors that underlie the associations uncovered.
